# Trial Versus No Trial of Spinal Cord Stimulation for Chronic Neuropathic Pain: Cost Analysis in United Kingdom National Health Service

**DOI:** 10.1111/ner.12898

**Published:** 2018-12-10

**Authors:** Rui V. Duarte, Simon Thomson

**Affiliations:** ^1^ Liverpool Reviews and Implementation Group University of Liverpool Liverpool UK; ^2^ Basildon and Thurrock University Hospitals Basildon UK

**Keywords:** Cost comparison, screening trial, spinal cord stimulation

## Abstract

**Objectives:**

The aim of the current project was to evaluate the spinal cord stimulation (SCS) screening trial success rate threshold to obtain the same cost impact across two identical sets of patients following either a prolonged screening trial prior to implantation strategy or a full implant without a screening trial.

**Materials and Methods:**

A cost impact analysis was carried out from a health care perspective and considered trial to implant rates reported in the literature. Items of resource use were costed using national averages obtained from the National Health Service (NHS) reference cost data base. Cost components were added up to derive total patient level costs for the NHS. Only the costs associated with the screening trial procedures and devices were considered.

**Results:**

The most conservative of our estimates suggest that a failure rate of less than 15% is cost saving to the NHS. A failure rate as high as 45% can also be cost saving if the less expensive nonrechargeable SCS devices are used. All the thresholds observed represent a considerably higher screening failure rate than that reported in the latest randomized controlled trials (RCTs) of SCS. A trial to implant ratio of 91.6% could represent savings between £16,715 (upper bound 95% CI of rechargeable implantable pulse generator [IPG] cost) and £246,661 (lower bound 95% CI of nonrechargeable IPG cost) per each 100 patients by adopting an implantation only strategy.

**Conclusions:**

Considerable savings could be obtained by adopting an implantation strategy without a screening trial. It is plausible that accounting for other factors, such as complications that can occur with a screening trial, additional savings could be achieved by choosing a straight to implant treatment strategy. Nevertheless, additional evidence is warranted to support this claim.

## INTRODUCTION

The prevalence of chronic pain in Europe is estimated to range between 13 and 51% 1, 2, which is considerably higher than that of other chronic conditions such as diabetes mellitus (type 1 or type 2), which has a much lower prevalence of 7% among men and 4.9% among women 3. Chronic pain with neuropathic characteristics has an estimated prevalence between 6.9 and 10% 4.

Spinal cord stimulation (SCS) is a recognized option for the management of chronic pain of neuropathic etiology. The effectiveness of SCS has been demonstrated for the management of neuropathic pain conditions such as failed back surgery syndrome (FBSS) 5, complex regional pain syndrome (CRPS) 6, and painful diabetic neuropathy 7.

Early clinical trials for SCS involved the use of trials of stimulation before permanent implantation of device. The aim was to help better identify patients who would benefit from the complete procedure—allowing physicians to assess the ability of the device to cover the patient's area of pain and the associated level of paraesthesia. Current guidance from the National Institute of Health and Care Excellence (NICE) on SCS for chronic pain of neuropathic or ischaemic origin is based on these trials 8. However, it stipulates that only patients who have undergone a successful trial of stimulation be allowed to have the device.

As the therapy and the devices have evolved, the percentage of patients receiving a definitive implant after the trial appears to have increased. Trial to implant rates observed in randomized controlled trials (RCTs) range from 67% in 2000 6, to 83% in 2007 5 and 86% in 2016 9. A low screening trial failure rate increases the total costs and decrease the value of the screening trial if only a small number of patients are considered as not benefiting from the treatment. Opinion also remains divided on the usefulness of a screening trial as a predictor of long‐term treatment effectiveness with reports of screening trials having resulted in the exclusion of good candidates for SCS 10. Trial stimulation may provide both false positive and false negative responses, which may not translate into the same long‐term outcome from a permanent SCS. However, the guidance still mandates the inclusion of a screening trial as part of the initial assessment prior to patients receiving a permanent device.

There is a need to evaluate the usefulness of a screening trial prior to the definitive implantation as a screening trial may result in increased health care resource use. The hypothesis underpinning this project is that it is more efficient to go directly to a definitive implant. The aim of the study is to estimate the percentage of successful trials at which point it would be more efficient not to carry out a screening trial prior to a definitive implant.

## MATERIALS AND METHODS

### Rapid Review of Economic Evaluations of SCS

A rapid review was conducted using the electronic data base MEDLINE to identify economic evaluations assessing the cost‐effectiveness of SCS for the management of neuropathic pain. The objective of the rapid review was to identify the costs associated with a SCS device procedure including the screening trial. The search strategy comprised the following combination of key words: 1) spinal cord stim*; 2) (economic OR cost*); 3) #1 AND #2.

The searches covered the period from the data base inception until 15th December 2017. Literature search results were uploaded to, and managed using EndNote X7.7.1 software.

### Data Extraction and Synthesis From Studies Identified in the Rapid Review

Relevant data concerning the screening trial period was extracted from the identified economic evaluations. Such data included bibliographic information (author(s) and year of publication), general information (country, population), screening trial characteristics (duration, setting of screening trial, key cost categories, and associated costs). A narrative synthesis was used to summarize and present the information provided in the selected articles.

### Decision Tree

**Figure 1:**
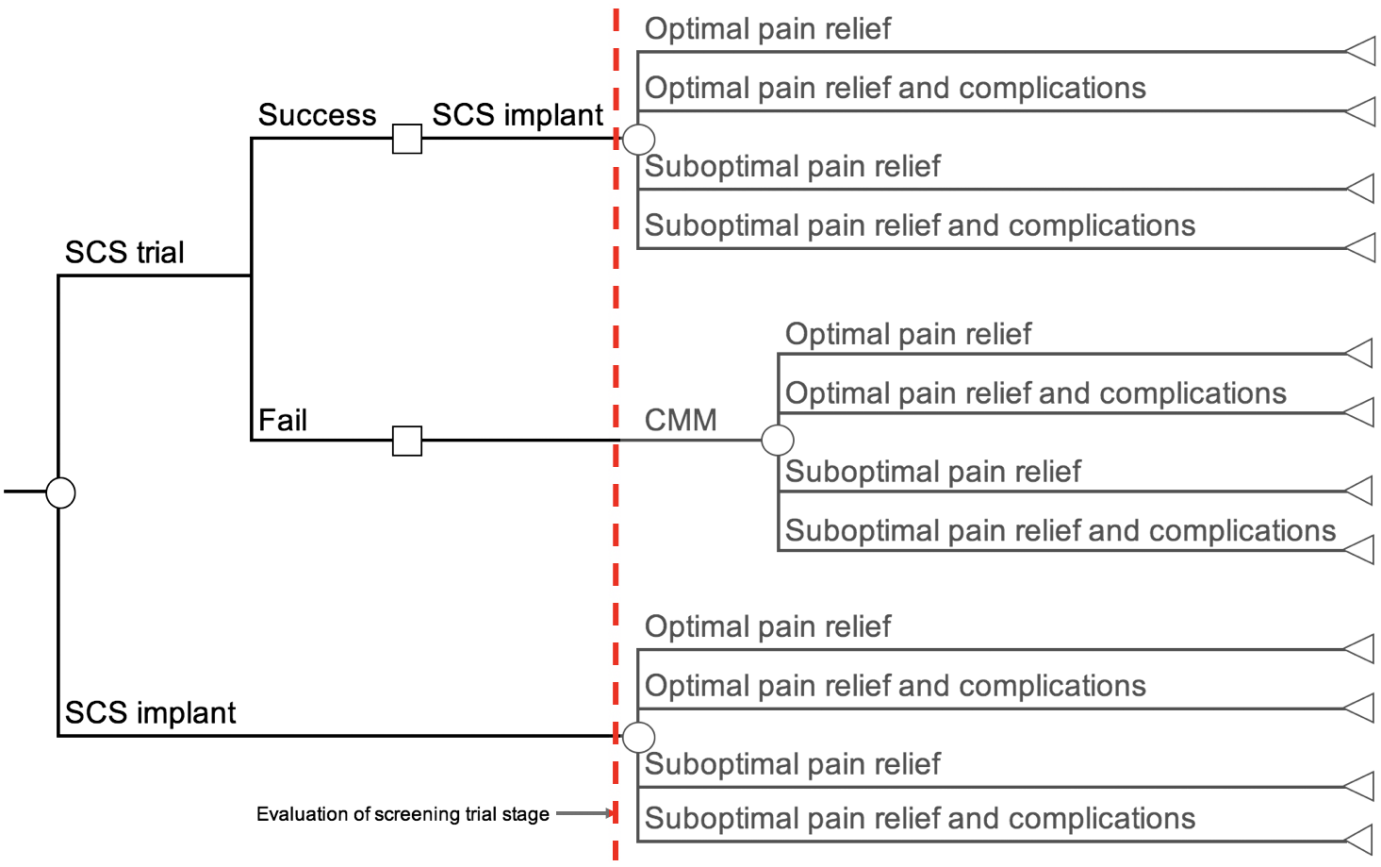
Decision tree for SCS trial strategy vs. SCS implant strategy. [Color figure can be viewed at wileyonlinelibrary.com]

A simple decision tree model was constructed in Microsoft Excel to compare the costs associated with two options: 1) SCS trial or 2) SCS implant (Fig. 1). The SCS trial strategy requires an initial screening trial in which patients that obtain satisfactory pain relief (i.e., success) will proceed to have a permanent SCS implant. Patients who do not obtain satisfactory pain relief (i.e., fail) will proceed to conventional medical management (CMM). The SCS implant strategy does not require the initial screening trial with the patients bypassing this stage and proceeding to an SCS implant. Patients receiving either SCS or CMM may obtain optimal or suboptimal pain relief, and additionally may experience complications. The scope of the current study is to evaluate the acute initial cost. Therefore, only the first part of the decision tree which evaluates the screening trial stage is considered.

### Costs

Items of resource use were costed using national averages obtained from national sources for the year 2015/2016 (NHS reference cost data base) 11. Cost components were added up to derive total patient level costs for the NHS with and without a screening trial prior to definitive implant of a SCS device.

### Data Analysis

A cost comparison between an implant only strategy and a trial and implant strategy was carried out from an NHS perspective. The scope of the current study is to evaluate the acute initial cost of these two strategies. Therefore, the analysis does not take into account other costs such as those associated with complications, infections, and post‐implant health care resource use.

The base‐case analysis takes into consideration a pathway using a nonrechargeable SCS device. A deterministic sensitivity analysis was carried out on the price of the device, taking into consideration rechargeable vs. nonrechargeable, and on the trial success rate to estimate potential savings that could be achieved if the extended trial period were not mandatory.

For contextualization purposes, we report the analyses considering different trial to implant ratios reported in RCTs and large case series assessing the use of SCS for neuropathic pain conditions.

## RESULTS

### Rapid Review of Economic Evaluations of SCS

**Figure 2:**
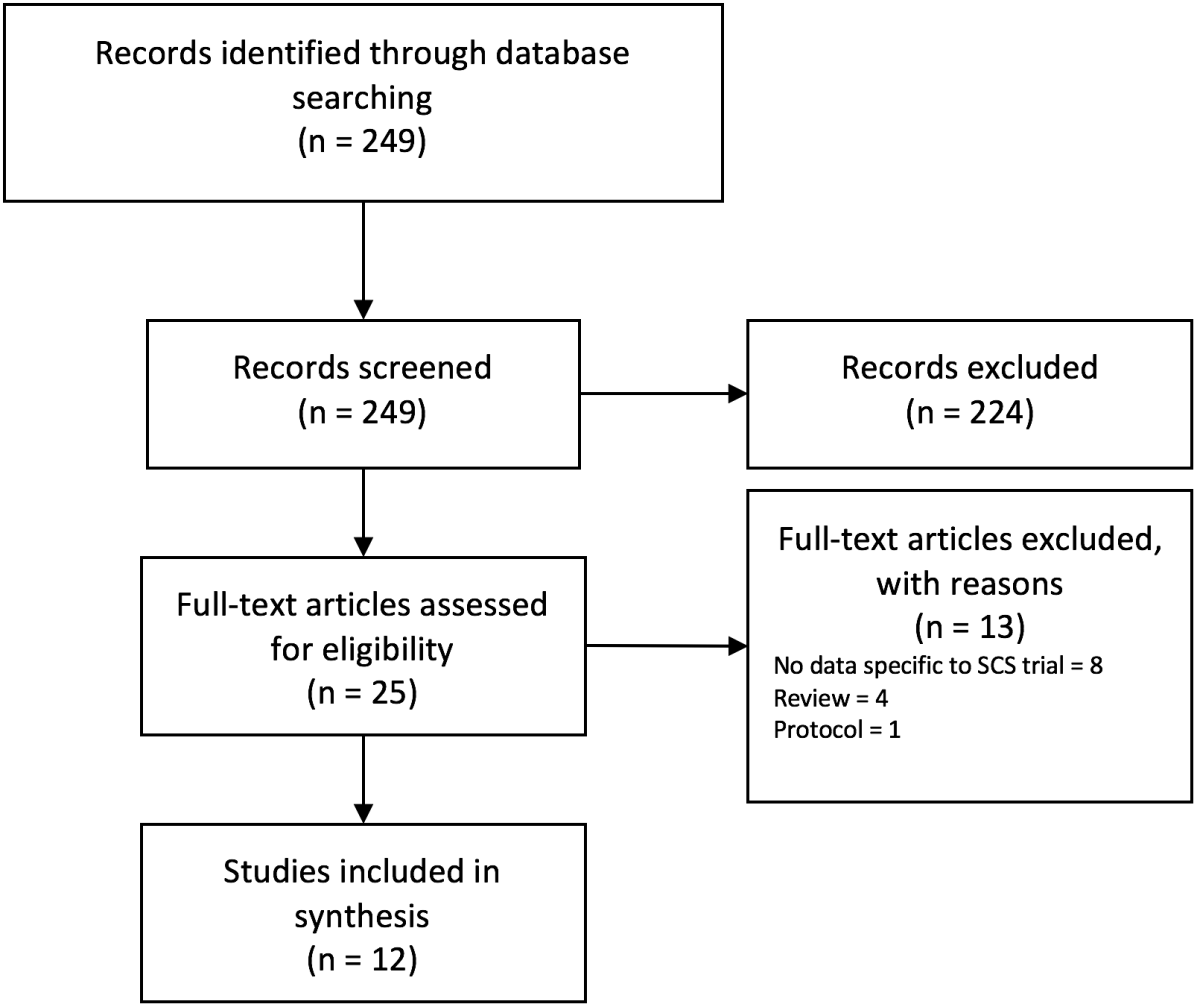
PRISMA flowchart detailing the study selection process.

The search resulted in a total of 249 records (Fig. 2). Following the screening stages of the review, data was extracted from 12 papers reporting 11 economic evaluations (Table 1). The majority of the economic evaluations (*n* = 7) assessed the cost‐effectiveness of SCS for a population of patients with FBSS, three studies included patients with CRPS, one study comprised both patients with FBSS and CRPS, while one encompassed patients with FBSS, CRPS, and other neuropathic pain conditions.

**Table 1:** Screening Trial Data in the Economic Evaluations Identified.

Author (year)	Country	Population	Duration (range)	Setting	Cost categories	Cost
Budd 12	UK	FBSS	Six days (4–7)*	Inpatient	Cost of implantation*	1157 UK £
Kemler and Furnee 13	The Netherlands	CRPS	NR	NR	Implant test lead	664 Euros
					Implant SCS system	8458 Euros
Kemler et al. 14	UK	CRPS	NR	NR	SCS screening trial	4069 UK £
					Failed screening electrode removal	1800 UK £
					IPG implantation†	9762 UK £
Kumar et al. 15	Canada	FBSS	NR	NR	Evaluation and implantation cost	16,936 CAN $
Kumar and Rizvi 16	Canada	FBSS	NR	NR	Pre‐implant	4120 CAN $
					Implant procedure	22,750 CAN $
		CRPS	NR	NR	Pre‐implant	4161 CAN $
					Implant procedure	23,226 CAN $
Hollingworth et al. 17	USA	Workers’ compensation recipients FBSS	NR	NR	Mean initial SCS procedure‡	21,282 US $
Hornberger et al. 18	USA	FBSS	NR	NR	Initial procedure (nonrechargeable)§	26,005 US $
					Initial procedure (rechargeable)§	35,109 US $
Mekhail et al. 19	USA	Neuropathic pain	NR	Outpatient	SCS trial	7248 US $
					SCS implant	19,687 US $
Simpson et al. 20	UK	FBSS and CRPS	NR	NR	Average cost per screen	4069 UK £
					Average cost of failed screening	1080 UK £
					Average cost of device implant	11,269 UK £
Manca et al. 21 and Taylor et al. 22	UK	FBSS	2.5 (1–5)	Inpatient	SCS screening trial	4442 UK £
					Failed screening electrode removal	1800 UK £
					IPG implantation†	9762 UK £
Zucco et al. 23	Italy	FBSS	NR	NR	Lead implantation	2335.8 Euros
					IPG implantation	5857.2 Euros

* Trial + implantation.

† Considering an IPG cost of 7761 UK £.

‡ Including 51 SCS trial procedures and 27 permanent device implants.

§ Includes physician and facility costs for the SCS trial.

CRPS, complex regional pain syndrome; FBSS, failed back surgery syndrome; IPG, implantable pulse generator; NR, not reported; SCS, spinal cord stimulation.

SCS screening trial details have not been commonly reported in the economic evaluations identified. The resource use categories that are considered when costing the screening trial also seem to vary, with some studies including the costs of the implantable pulse generator (IPG) with the screening. Due to the variability in costing strategies of the screening trial period, it was decided to use the costs employed in Health Technology Appraisals (HTAs) 20, 24 that informed or are in the process of informing recommendations for the use of SCS in England.

### Resource Use Costs

The cost of implantation of a SCS device for pain management and the cost of a screening trial was retrieved from the NHS reference cost data base (Table 2). However, the NHS reference cost data base does not include the cost of electrode removal following a failed screening trial. The cost associated to a failed screening trial was derived from Simpson et al. 20 and adjusted to the 2016 price year (Table 3). This cost was chosen as it was used to inform NICE guidance on SCS 8. SCS devices are purchased via the NHS Supply Chain catalogue with significant discounts for the NHS. Alternatively, locally tendered contracts may be used to secure additional discounts for a commitment to purchase a certain volume from a manufacturer. Due to the possibility of different costs associated with the IPG, for this exercise the costs for a nonrechargeable and for a rechargeable IPG presented in a recent technology appraisal submitted to NICE were used 24. The costs for implantation include the cost of device which taking into account the cost for HRG AB12Z could range from £1692 to £7320 for a nonrechargeable IPG and from £6530 to £15,222 for a rechargeable IPG.

**Table 2:** National Schedule of Reference Costs Year 2016–2017.

Currency	Currency description	Unit cost
AB12Z	Insertion of neurostimulator for pain management	£7196.49
AB14Z	Insertion of neurostimulator electrodes for pain management	£3006.94

**Table 3:** Resource Use Costs Employed for the Current Exercise.

Item of resource use	Cost (95% CI)	Source
Implantation (nonrechargeable)*	£11,281.00 (£8888.00–£14,516.00)	Willits et al. (2017) 24
Implantation (rechargeable)*	£17,422.00 (£13,726.00–£22,418.00)	Willits et al. (2017) 24
Cost per trial	£3006.94	NHS reference costs
Failed screening (electrode removal)	£2133.81	Simpson et al. (2009) 20

All costs updated to 2016 prices.

* Cost of implantation includes technology costs and procedural costs.

The average total cost for a patient following each of the possible pathways presented in Figure 1 would be as follows:

- Total cost successful trial and implant (nonrechargeable) = £14,288
- Total cost successful trial and implant (rechargeable) = £20,429
- Total cost failed trial (nonrechargeable or rechargeable) = £5141
- Total cost implant only strategy (nonrechargeable) = £11,281
- Total cost implant only strategy (rechargeable) = £17,422

### Base‐Case Results

The total cost to the NHS of 100 patients undergoing a successful SCS trial followed by implant of a nonrechargeable IPG would be £1,428,794. The total cost to the NHS of 100 patients going straight to an implant of a nonrechargeable IPG would be £1,128,100, the equivalent to a saving of £300,694 per 100 patients.

The point at which both strategies would have equivalent costs occurs at the point where 33 patients (or 33%) fail the screening trial. This is the minimum failure rate after which a screening trial strategy becomes cost saving. This is a higher screening failure rate than observed in recent RCTs and large case series (Table 4). Consideration of trial to implant ratios reported suggests that a screening trial strategy would only result in cost savings based on the study by Kemler et al. 6. Potential savings by adopting an implant only strategy range between £7984 and £227,517.

**Table 4:** Comparison of Costs Accounting for Trial to Implant Rates Reported in the Literature.

Source	Design	Trial to implant rate (%)	Costs for nonrechargeable IPG	Cost difference for nonrechargeable IPG
Base‐case*	–	–	£1,128,100	–
Kemler et al. 6	RCT	67	£1,126,937	−£1163
Kumar et al. 5	RCT	83	£1,273,292	£145,192
Kapural et al. 9	RCT	86	£1,300,733	£172,633
Hayek et al. 25	Case series	68	£1,136,084	£7984
Thomson et al. 26	Case series	92	£1,355,617	£227,517

* Considers 100 patients going straight to SCS implant.

IPG, implantable pulse generator; RCT, randomized controlled trial.

### Deterministic Sensitivity Analysis

#### Nonrechargeable IPG

The difference between the total cost of the possible pathways considering 100 patients receiving a SCS implant following a successful screening trial taking into account the 95% CIs reported for the costs of implantation of a nonrechargeable SCS device, is the same as in the base‐case. However, there would be a change in the point at which the SCS screening trial becomes cost saving (i.e., the cost of the device dictates when cost savings occur).

Considering the cheapest estimate of the cost of a nonrechargeable IPG (lower bound of the 95% CI), equivalent costs between the strategies would be observed if 45 out of 100 patients would fail the SCS screening trial. The upper bound of the 95% CI indicates that equivalent costs between the strategies would occur at a trial to implant ratio of 75.7%.

Consideration of reported trial to implant ratios suggests that a screening trial strategy would only result in cost savings when considering an upper bound cost for a nonrechargeable IPG in two cohorts (Table 5).

**Table 5:** Comparison of Costs for a Rechargeable IPG Accounting for Trial to Implant Rates Reported in the Literature.

Source	Design	Trial to implant rate (%)	Costs for rechargeable IPG	Cost difference for rechargeable IPG
Base price*	–	–	£1,742,200	–
Kemler et al. 6	RCT	67	£1,538,384	−£203,816
Kumar et al. 5	RCT	83	£1,782,995	£40,795
Kapural et al. 9	RCT	86	£1,828,859	£86,659
Hayek et al. 25	Case series	68	£1,553,672	−£188,528
Thomson et al. 26	Case series	92	£1,920,589	£178,389

* Considers 100 patients going straight to SCS implant.

IPG, implantable pulse generator; RCT, randomized controlled trial.

#### Rechargeable SCS

The higher cost of a rechargeable SCS device leads to equivalent costs between the strategies being reached with a smaller number of patients failing the SCS screening trial. The total cost to the NHS of 100 patients undergoing a successful SCS trial followed by implant of a rechargeable IPG would be £2,042,894. The total cost to the NHS of 100 patients going straight to an implant of a rechargeable IPG would be £1,742,200, the equivalent to a saving of £300,694 per 100 patients.

The point at which equivalent costs would be observed between the strategies at a base price of £17,422 per rechargeable SCS device would occur at the point where 20 out of 100 patients fail a screening trial of SCS. Considering trial to implant ratios in more recent RCTs, savings between £40,795 and £86,659 per each 100 patients could be obtained by adopting a straight to SCS implant strategy (Table 6).

**Table 6:** Comparison of Costs Accounting for Trial to Implant Rates Reported in the Literature and 95% CIs for a Nonrechargeable IPG.

Source	Design	Trial to implant rate (%)	95% CI lower bound costs	Cost difference 95% CI lower bound costs	95% CI upper bound costs	Cost difference 95% CI upper bound costs
Current study*	–	–	£888,800	–	£1,451,600	–
Kemler et al. 6	RCT	67	£966,606	£77,806	£1,343,682	−£107,918
Kumar et al. 5	RCT	83	£1,074,673	£185,873	£1,541,797	£90,197
Kapural et al. 9	RCT	86	£1,094,935	£206,135	£1,578,943	£127,343
Hayek et al. 25	Case series	68	£973,360	£84,560	£1,356,064	−£95,536
Thomson et al. 26	Case series	92	£1,135,461	£246,661	£1,653,237	£201,637

* Considers 100 patients going straight to SCS implant.

CI, confidence interval; IPG, implantable pulse generator; RCT, randomized controlled trial.

The point at which equivalent costs between the strategies would be achieved considering the lower bound of the 95% CI on the price of a rechargeable SCS device would occur when 26% of patients fail the screening trial. The upper bound of the 95% CI suggests this to occur when 15 out of 100 patients fail the SCS screening trial. This is the most approximate to the proportions of patients failing the screening trial reported in the most recent RCTs (Table 7). A SCS implant only strategy could result in savings between £16,715 and £138,421 per each 100 patients when compared with a screening trial strategy.

**Table 7:** Comparison of Costs Accounting for Trial to Implant Rates Reported in the Literature and 95% CIs for a Rechargeable IPG.

Source	Design	Trial to implant rate (%)	95% CI lower bound costs	Cost difference 95% CI lower bound costs	95% CI upper bound costs	Cost difference 95% CI upper bound costs
Current study*	–	–	£1,372,600	–	£2,241,800	–
Kemler et al. 6	RCT	67	£1,290,752	−£81,848	£1,873,116	−£368,684
Kumar et al. 5	RCT	83	£1,476,227	£103,627	£2,197,663	−£44,137
Kapural et al. 9	RCT	86	£1,511,003	£138,403	£2,258,515	£16,715
Hayek et al. 25	Case series	68	£1,302,600	−£70,256	£1,893,400	−£348,400
Thomson et al. 26	Case series	92	£1,580,557	£207,957	£2,380,221	£138,421

* Considers 100 patients going straight to SCS implant.

CI, confidence interval; IPG, implantable pulse generator; RCT, randomized controlled trial.

## DISCUSSION

The current study observed that savings could be made to the NHS and potentially other European health services if a screening trial was not mandatory prior to SCS implantation for the management of chronic neuropathic pain. SCS is considered a cost‐effective option for this population when compared to CMM or re‐operation (in the case of patients with FBSS) 20, 21, 22. However, areas where additional savings could be made are important to ensure that patients continue to have access to this technology.

The disparities between trial to implant ratios used to assess the potential impact of not having a screening trial prior to IPG implantation may be due to differences in eligibility criteria for SCS used in the trials and between countries. A rate of 67% has been observed in an RCT of SCS for CRPS 6, while for FBSS, an RCT has reported a trial to implant ratio of 83% (considering the number of patients that achieved 50% or more leg pain relief or 80% paraesthesia coverage during the screening trial and not including those patients that requested to be implanted with a device despite failing the screening trial) 5. Recent service reviews conducted in the US and in the UK reported trial to implant ratios of 68% 25 and 92% respectively 26. A trial to implant rate of 91% was observed in data from the Netherlands, and therefore similar to that observed in the UK 27. Analysis of insurance claim data bases in the US report trial to implant rates that range from as low as 41.4% 28, up to 64.7% 29.

In the UK, NICE Technology Appraisal 159 expresses criteria that patients must meet prior to receiving approval for SCS funding 8. This includes a multidisciplinary assessment and a screening trial of SCS. The multidisciplinary team is not defined, but in the UK it is interpreted as a pain physician, psychologist, and other pain management experienced members including physiotherapist and nurse 30. In the authors practice (ST) this is a dynamic educative process that addresses the needs, timing, and suitability for SCS. Assessment of eligibility varies across Europe with countries such as Belgium employing a multidisciplinary assessment, while in Germany and the Netherlands a psychological evaluation is performed 31. Not all facilities have access to a multidisciplinary pain team. In USA a psychology opinion is usually required 32, but the use of a multidisciplinary team in the selection of a patient for SCS is variable. Although common practice, it is unclear if patients are refused a trial of SCS based on a psychological evaluation 33. It has been suggested that in the USA the psychological assessment is simply used as a means of ensuring approval by third‐party payers 34.

Clinical diagnosis may also have an effect in the trial to implant ratios reported for SCS. The trial to implant ratios presented within this assessment were based on studies where the diagnosis was specific, i.e., CRPS only 6, FBSS only 5, or comprising patients with heterogeneous clinical diagnoses 9, 25, 26. RCTs evaluating SCS for painful diabetic neuropathy have reported trial to implant ratios of 77% 35 and 92.5% 36. In addition to clinical diagnosis, it has been suggested that the presence of brush‐evoked allodynia may result in patients having a lower chance of achieving successful pain reduction with SCS when compared with patients without allodynia 37.

The current study is a theoretical exercise and its results should be interpreted with caution due to inherent limitations. Candidates for SCS are more susceptible to possible infections and complications if having to undertake a screening trial 38. The costs associated with these potentials adverse events have not been taken into consideration in the current report but would undoubtedly add to the costs of a screening trial, further indicating that a strategy with a screening trial prior to full implant may not be a cost‐effective strategy. Instrumenting the epidural space on two occasions if using a screening trial prior to full implant doubles the less common risks of dural puncture and neurological harm. The clinical utility and cost‐effectiveness of screening trials of SCS is currently being investigated in the National Institute for Health Research funded TRIAL‐STIM RCT 39.

Another consideration that needs to be taken into account is the long‐term effectiveness of SCS. Nevertheless, available evidence has suggested that screening trials may not be predictive of long‐term success and may actually exclude good candidates for SCS 10. Therefore, explantation costs that may be expected due to ineffectiveness of SCS may not actually occur, as the trial may not be a good predictor of SCS outcome. To highlight this point, one service review that reported a trial to implant ratio of 91.6% was associated with an explant rate of 6.7% 26, while another service review with a trial to implant rate of 67.8% resulted in an explant rate of 23.9% 25.

In conclusion, while there is no consensus as to the value of a screening trial to identify those patients that would most benefit from SCS, there are potential savings that could be made by not undertaking a trial prior to SCS implantation. The use of recently observed trial to implant failure rates suggests that these savings can be substantial, therefore making SCS an even more attractive technology to health care commissioners.

## Authorship Statements

Rui V. Duarte and Simon Thomson conceptualized the study. Rui V. Duarte conducted the acquisition, analysis, and interpretation of the data. Rui V. Duarte prepared the manuscript draft with input from Simon Thomson. All authors approved the final manuscript.

## COMMENTS

I have seen the comments and the changes of the authors. I disagree with the “all in one policy” advocated by many companies nowadays. For some indications this might end up in many failures (up to 40%). But if Neuromodulation has a different view on this problem, accept this manuscript with an editorial comment.

Maarten van Kleef, MD, PhD

*Maastricht, The Netherlands*

***

It is interesting to focus up the question of trial vs. no trial. It could be useful to combine results from other European countries in order to provide more satisfactory conclusions to be used by pain physicians.

Pasquale De Negri, MD

*Basilicata, Italy*

***

The literature on neuromodulation has only begun to address the many variations in regional practices, patient and practitioner attributes, and technology from a health care economic perspective. The value of this paper is not only that it adds to the literature but also that it clearly acknowledges its scope ‐ e.g., acute initial cost only, payor perspective, NHS practices (trial electrodes anchored for conversion to permanent implant), rapid, limited review of literature ‐ and thus the limits of its generalizability. The authors prudently point out that “The current study is a theoretical exercise and its results should be interpreted with caution.” In particular, the high implant‐to‐trial ratios in the publications they have tabulated might not be representative of everyday clinical practice, which (as reflected in one of their references) can be below 50% (1).

This otherwise carefully written paper contains multiple references to “trial to implant ratio” when in fact the inverse (implant to trial ratio) clearly is intended; all values are below 100%. I point out this error now with apologies that I overlooked it on initial reading: it is so common in everyday usage as to pass notice, and now it is too late in the publication process to correct it. In any event, until and unless implant to trial to ratios become 100%, and the point becomes moot, this humble reviewer will keep Tolstoy in mind: “A man is like a fraction whose numerator is what he is and whose denominator is what he thinks of himself.”

Reference

1. Huang KT, Martin J, Marky A et al. A national survey of spinal cord stimulation trialto‐permanent conversion rates. Neuromodulation 2015;18:133–139.

Richard North, MD

*Baltimore, MD, USA*

Comments not included in the Early View version of this paper.
